# Annotations

**Published:** 1899-11-25

**Authors:** 


### ANNOTATIONS.

### Overcrowding on Foreign Vessels.

The half-yearly report of Dr. Collingridge, medical officer for the Port of London, again serves to show the great variety of duties which fall upon him and the importance of the sanitary work which is being carried out on the river, not only in protecting London from the importation of infectious disease, but in maintaining the health of that large population which depends on the river for its livelihood. Among the many points dealt with there is one to which especial attention may be drawn, namely, the amount of overcrowding which is sometimes found in foreign vessels arriving in the port. The steamship "Jyden," of Randers, arrived in London from Libau with 39 passengers, all of whom were berthed in the port alleyway, the dimensions of this space being 20 feet by 6 feet 9 inches, giving roughly 45 cubic feet of air space per head. The Danish steamer "Magnus," from Libau, arrived with 176 foreign emigrants, 169 of whom were carried in No. 3 'tween deck, in a space which allowed only 50·02 cubic feet, and only 6·15 square feet per head. Men, women, and children were all berthed together, and it was found that one death and one birth had occurred among these people on the voyage, the dead body remaining until it could be removed in London. It is with this sort of thing that the English carrying trade has to compete, and our efforts to enforce some reasonable standard of decency on board English vessels meet with but little encouragement from passengers of this sort. It unfortunately seems to be the fact that among emigrants the one thing asked for is cheapness. There is no degree of filth and discomfort which they will not accept so only that they are carried to their new homes cheaply.

### The Specialist and His Telegrams.

Perhaps it is because the specialist is a modern product that his bent is so strongly towards modern ways, and towards utilising for his own purposes the facilities offered by modern social methods. That he should be a man of many instruments is not surprising, and that these instruments should run largely to electricity is only in the nature of things; that his letters should be typed and his notes dictated to a shorthand writer may be taken for granted; and that he should be on the telephone, and have a registered telegraphic address are mere matters of course. This telegraphic address is of peculiar interest, for it enables the specialist to do what without it he would find difficult; namely, to announce his line of practice. No one would care to put on his door-plate, "Specialist in Heart Disease," but nothing is simpler than to stamp one's notepaper, "Telegrams—'Cardiac, London,'" which serves the same purpose. Moreover, this is just the sort of thing the public likes. There is something so "simple" and "common sense" about it. It really is becoming a serious matter to the well-to-do public, who of course never write letters but telegraph for everything, to have to decide what doctor to call in! But this telegraphic address business is the very thing. Why bother about knowing the names of all the specialists? If one knows one's own complaint that is quite enough; and every person of intelligence, of course, knows what is the matter with him, notwithstanding the fuss that pedantic doctors make about what they call diagnosis! All that one has to do when one feels "chippy" is to decide what sort of a man one wants, and then to send for him. Is it headache that we suffer from? "Headache, London," will fetch us just the specialist we want. Or, if we feel doubtful about our liver, "Liver, London," will do the trick. Who the man is, who, as they say of dogs, "answers to the name," what he has done, or even what his real name is, we do not know; perhaps we do not care. Why, indeed, should we trouble about all these details? It is the specialist that we want, and by this ingenious device we get him. Perhaps the specialist also gets what he wants, so that the accommodation is quite mutual. Which ought to be satisfactory to all parties—except those who have been too late in choosing their registered telegraphic address.

### Early Rising.

"Blessed be the man that invented sleep" said Sancho Panza; and a subsequent philosopher improved on the sentiment, thus:—

> "Blest be the man who first invented sleep,
> But cursed be he, with curses long and deep,
> Who first invented, and then went advising,
> That artificial cut-off, early rising."

The sentiment is one that appeals to unregenerate human nature; but in face of the copy-book maxims that describe early rising as something not far below the cardinal virtues, most of us have hitherto had some hesitation in avowing our distaste for seeing the sun rise, or taking a five-mile walk before breakfast, or performing any other of those feats which tend to make man, as Sydney Smith said, "conceited all the morning and sleepy all the afternoon." But now science has come to the support of unregenerate man, and, according to an article which appeared some time ago in the *British Medical Journal*, early rising does not make for health, but for disease. But, of course, it must be remembered that early rising is a comparative thing. If one goes to bed at eight, like Jules Verne, one may risk rising at five in the morning, like that veteran novelist. But people vary, either from habit or constitution, as to the time when they sleep best. With some the most refreshing sleep is obtained before midnight, while others cannot close their eyes before the small hours of the morning. Clearly, therefore, early rising means a different thing for these two, and to compel them to follow the same rule is to punish them, and probably to injure their health. The main point is to have enough of sleep, at whatever hour we take it, and it is well to get rid of any notion of superior virtue or even activity to be gained by stinting oneself in that very real necessary of life. Even activity we say, for it is quite possible to be "up" and not "doing," and a good many of those who make a boast of their early rising seem to think that it is an end in itself, instead of only a means to the accomplishment of some definite object. Perhaps the inveterate early riser, who finds his highest enjoyment in telling all his acquaintance that five hours' sleep is enough for any man—he never takes more—perhaps this man can hardly be cured of the vice; but, at least, if we are able to assure him, on the authority of the *British Medical Journal*, that he is likely to produce "hardening of the arteries and the less ready action of the vaso-motor system," we may be able to make a better fight against the accusations of laziness which he loves to cast at all who do not share his very uncomfortable hobby.

